# Recurrent *HOXB13* mutations in the Dutch population do not associate with increased breast cancer risk

**DOI:** 10.1038/srep30026

**Published:** 2016-07-18

**Authors:** Jingjing Liu, Wendy J. C. Prager–van der Smissen, Marjanka K. Schmidt, J. Margriet Collée, Sten Cornelissen, Roy Lamping, Anja Nieuwlaat, John A. Foekens, Maartje J. Hooning, Senno Verhoef, Ans M. W. van den Ouweland, Frans B. L. Hogervorst, John W. M. Martens, Antoinette Hollestelle

**Affiliations:** 1 Department of Medical Oncology, Erasmus MC Cancer Institute, Wytemaweg 80, 3015 CN Rotterdam, The Netherlands; 2 Division of Molecular Pathology, Netherlands Cancer Institute, Plesmanlaan 121, 1066 CX Amsterdam, The Netherlands; 3 Department of Clinical Genetics, Erasmus University Medical Center, Wytemaweg 80, 3015 CN Rotterdam, The Netherlands; 4 Division of Diagnostic Oncology, Netherlands Cancer Institute, Plesmanlaan 121, 1066 CX Amsterdam, The Netherlands; 5 Cancer Genomics Netherlands, Universiteitsweg 100, 3584 CG Utrecht, The Netherlands

## Abstract

The *HOXB13* p.G84E mutation has been firmly established as a prostate cancer susceptibility allele. Although *HOXB13* also plays a role in breast tumor progression, the association of *HOXB13* p.G84E with breast cancer risk is less evident. Therefore, we comprehensively interrogated the entire *HOXB13* coding sequence for mutations in 1,250 non-*BRCA1/2* familial breast cancer cases and 800 controls. We identified two predicted deleterious missense mutations, p.G84E and p.R217C, that were recurrent among breast cancer cases and further evaluated their association with breast cancer risk in a larger study. Taken together, 4,520 familial non-*BRCA1/2* breast cancer cases and 3,127 controls were genotyped including the cases and controls of the whole gene screen. The concordance rate for the genotyping assays compared with Sanger sequencing was 100%. The prostate cancer risk allele p.G84E was identified in 18 (0.56%) of 3,187 cases and 16 (0.70%) of 2,300 controls (OR = 0.81, 95% CI = 0.41–1.59, *P* = 0.54). Additionally, p.R217C was identified in 10 (0.31%) of 3,208 cases and 2 (0.087%) of 2,288 controls (OR = 3.57, 95% CI = 0.76–33.57, *P* = 0.14). These results imply that none of the recurrent *HOXB13* mutations in the Dutch population are associated with breast cancer risk, although it may be worthwhile to evaluate p.R217C in a larger study.

Breast cancer is the second leading cause of cancer death in Western countries and the most frequently diagnosed cancer in Western women. A family history of breast cancer is a major risk factor for developing breast cancer. Approximately 10–15% of breast cancer patients have at least one first-degree relative with breast cancer. Depending on the number of affected first-degree relatives, this implies risk ratios for breast cancer of 1.80 for one affected relative to 3.90 for three or more affected relatives^1^.

Familial breast cancer has been associated with mutations in several high- and moderate-risk breast cancer susceptibility genes, as well as an increasing number of low-risk breast cancer susceptibility alleles. The two high-risk genes *BRCA1* and *BRCA2* were identified in the 1990s and germline mutations in these genes confer average cumulative lifetime breast cancer risks by age 70 of 65% and 45%, respectively^2^,^3^,^4^. Mutations in moderate-risk genes *ATM, CHEK2, PALB2, and RAD50* confer 2- to 4-fold increased breast cancer risks^5^, although recent evidence suggests that the breast cancer risk conferred by *PALB2* mutations may be higher than initially thought^6^,^7^. The more than 90 identified common low-risk alleles, on the other hand, display small effect sizes (*i.e.* per allele odds ratios) of up to 1.3^8^. However, taken together in a polygenic risk score (PRS; calculated from 77 SNPs) the lifetime risk of breast cancer for women in the highest quantile of this PRS was 17%^9^. In total, these breast cancer susceptibility genes and alleles account for approximately 35% of the familial breast cancer risk, which means that the underlying cause of the majority of the familial breast cancer risk thus still remains unexplained.

In this respect, the rare variant c.251G > A (p.G84E; rs138213197) in the *HOXB13* gene was reported to be associated with prostate cancer^10^. Meta-analyses have estimated the increased prostate cancer risk from this mutation to be 4- to 5-fold and even higher among early onset prostate cancer patients and prostate cancer patients with a family history of prostate cancer^11^,^12^,^13^,^14^. Moreover, fine-scale mapping at the *HOXB* gene cluster at 17q21–22 had identified a number of highly correlated common SNPs that were associated with prostate cancer risk and tagging the rare *HOXB13* p.G84E variant. This not only further established the association between *HOXB13* p.G84E and prostate cancer risk, but also provided evidence that GWAS associations could actually be driven by rare variants^15^. Interestingly, the *HOXB13* gene encodes a transcription factor that plays an important regulatory role during embryonic development, but also in tumorigenesis. For example, *HOXB13* was reported to regulate the transcription of androgen receptor (AR) target genes^16^ and together with *HOXA9, HOXB13* is the most commonly deregulated gene in solid cancers^17^. Moreover, *HOXB13* was shown to preferentially bind a low-risk prostate cancer susceptibility allele located in an AR and FOXA1 binding site (*i.e.* rs339331), thereby enhancing *RFX6* expression and promoting metastasis^18^.

In breast cancer, *HOXB13* gene expression is regulated by estrogen in an ER dependent manner^19^. Furthermore, a high *HOXB13*:*IL17BR* expression ratio was found to be a prognostic and predictive biomarker for ER-positive breast cancer patients^20^,^21^. The poor response to tamoxifen therapy that is predicted from high HOXB13 expression has been shown to be mediated by HOXB13 through the direct suppression of ER, the induction of IL6 expression and mTOR pathway activation^22^. Considering these observations, *HOXB13* might also be a likely candidate for being a breast cancer susceptibility gene. So far, three studies have investigated this hypothesis but obtained contradictory results. In the study by Alanee *et al*.^23^, the *HOXB13* p.G84E mutation was shown to confer an increased breast cancer risk, however, Akbari *et al*. could not replicate this association in a larger study^24^. Laitinen *et al*. also found no association with breast cancer risk, but did observe a suggestive association in a particular high-risk subgroup^25^. Importantly, all three studies only investigated the prostate cancer risk-associated variant p.G84E.

In this study, we therefore analyzed the entire coding region of the *HOXB13* gene in 1,250 Dutch familial breast cancer cases and 800 geographically matched controls to establish whether the p.G84E mutation or other mutations in the *HOXB13* gene are associated with an increased breast cancer risk.

## Results

### *HOXB13* whole gene screen

**Figure 1:**
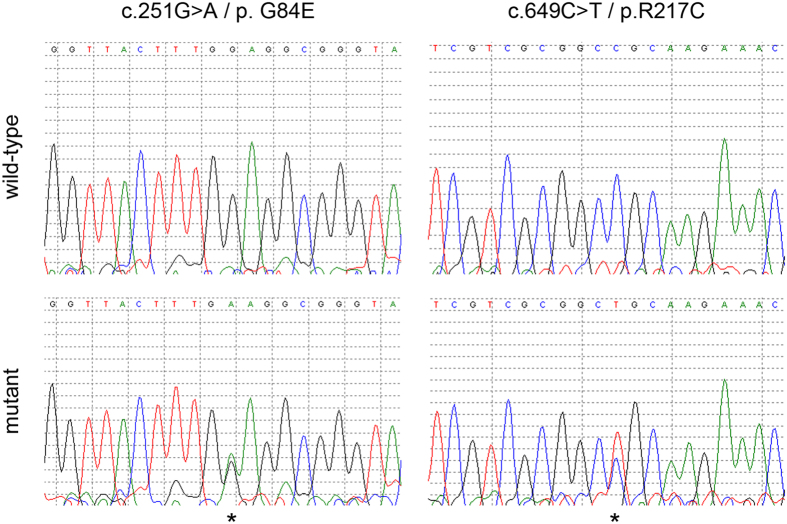
Identification of the c.251G > A (p.G84E) and c.649C > T (p.R217C) mutations. The lower electropherograms show the c.251C > A (left) and the c.649C > T (right) mutations which are indicated with an asterisk as compared with the wild-type sequences in the top panels.

We evaluated the entire coding sequence of the *HOXB13* gene for germline mutations in 1,250 non-*BRCA1*/*2* breast cancer patients and 800 controls from the Rotterdam Breast Cancer Study (RBCS) study. Using PCR and Sanger sequencing, we identified a total of eleven different rare variants (Table 1) and two more common variants (c.366C > T; p.S122S; rs8556; minor allele frequency (MAF) cases = 0.126; MAF controls = 0.138 and c.513T > C; p.S171S; rs9900627; MAF cases = 0.079; MAF controls = 0.091). Seven of the eleven rare variants were missense variants and five of these were present either in only one case or one control. The other two missense variants (*i.e.* c.251G > A and c.649C > T) were detected in multiple cases and controls (Fig. 1). The c.251G > A (p.G84E) mutation was detected in 4 of 1,215 (0.33%) cases and 6 of 759 (0.79%) controls, whereas the c.649C > T mutation was detected in 6 of 1,206 (0.50%) cases and 1 of 765 (0.13%) controls (Table 1). For all identified missense variants the carrier frequency was low, resulting in insufficient power to draw meaningful statistical inferences from this sample size. However, it did appear that the prostate cancer risk variant p.G84E was less prevalent in breast cancer cases than controls, whereas the prevalence of the p.R217C variant appeared to be higher in breast cancer cases compared with controls. Interestingly, both missense mutations were predicted to be deleterious based on three different prediction classification tools: PredictSNP^26^ (*i.e.* 87% for p.G84E and p.R217C), Meta-SNP^27^ (*i.e.* 0.730 for p.G84E and 0.895 for p.R217C) and PON-P2^28^ (*i.e.* 0.967 for p.G84E and 0.974 for p.R217C). Moreover, p.G84E is localized in the MEIS binding domain, whereas p.R217C is localized to the homeodomain of HOXB13, further increasing the likelihood that these mutations are pathogenic^10^. For these reasons, we decided to further pursue these two variants in a second sample set by expanding RBCS (*i.e.* to all indexes from families counselled between 1994 and 2014 for cases and between 1996 and 2010 for controls) and by including the Amsterdam Breast Cancer Study (ABCS-F).

### Genotyping *HOXB13* p.G84E and p.R217C

In order to facilitate fast and accurate screening of the p.G84E and p.R217C mutations, two custom-designed Taqman genotyping assays were developed for analyzing all samples from the RBCS and ABCS-F case-control studies. In total, all 4,520 non-*BRCA1/2* breast cancer patients and 3,127 controls were genotyped. These also included the 1,250 non-*BRCA1*/*2* breast cancer patients and 800 controls from the RBCS study that were used in the whole gene screen to evaluate the quality of the genotyping assay. The concordance between the results from the custom-designed Taqman genotyping assay and Sanger sequencing of these patients was 100%. Interestingly, the p.G84E mutation was identified in 18 (0.56%) of 3,187 cases and 16 (0.70%) of 2,300 controls (Table 2). Consistent with the results from the whole gene screen, the p.G84E mutation was more prevalent in controls than cases, however, this was not statistically significant (OR = 0.81, 95% CI = 0.41–1.59, *P* = 0.54). The p.R217C mutation was identified in 10 (0.31%) of 3,208 cases and 2 (0.087%) of 2,288 controls (Table 2). Consistent with the results of the whole gene screen, the p.R217C mutation was more prevalent in cases than in controls, but this difference was not significant (OR = 3.57, 95% CI = 0.76–33.57, *P* = 0.14). These results imply that none of the recurrent *HOXB13* mutations in the Dutch population are associated with breast cancer risk.

## Discussion

The *HOXB13* c.251G > A (p.G84E) mutation has been shown to confer a 4- to 5-fold increased prostate cancer risk^11^,^12^,^13^. In this study, we have explored whether *HOXB13* gene mutations are also associated with breast cancer risk. Our results show that the prostate cancer risk variant p.G84E is not associated with breast cancer risk. Furthermore, another recurrent mutation in the *HOXB13* gene (*i.e.* c.649C > T; p.R217C) was also not associated with increased breast cancer risk, although it was more prevalent in cases than controls.

Interestingly, Alanee *et al*. had previously shown that the *HOXB13* p.G84E mutation conferred a moderate to high breast cancer risk^23^. The mutation was found in 6 (0.7%) of 877 familial, mostly Caucasian, non-BRCA1/2 breast cancer cases, while the frequency in controls was 0.1% (OR = 5.7, 95% CI = 1.0–40.7, *P* = 0.02). However, in a larger study (*i.e.* 4,037 cases of which 1,082 familial and 2,762 controls) conducted by Akbari *et al*., no association of the p.G84E mutation with breast cancer risk was observed among Canadian and Polish women of European origin^24^. The mutation was identified in 7 (0.17%) of 4,037 cases and 4 (0.14%) of 2,762 controls (OR = 1.2, 95% CI = 0.3–4.1, *P* = 1.0). Also a third study by Laitinen *et al*. consisting of 986 cases (*i.e.* of which 323 familial non-*BRCA1*/*2* and 663 unselected) and 1,449 controls found no overall association between the p.G84E mutation and (familial) breast cancer risk among Finnish women^25^. However, the authors did observe a suggestive association in a specific high-risk familial subgroup (*i.e.* 86 cases from the Pirkanmaa area of Finland; OR = 3.2, 95% CI = 0.9–11.9). Here in this study, we also did not observe an increased breast cancer risk associated with the p.G84E mutation in a relatively large study of 3,270 familial non-*BRCA1/2* breast cancer cases and 2,327 controls (OR = 0.81, 95% CI = 0.41–1.59, *P* = 0.54). It thus appears that the *HOXB13* p.G84E mutation is not associated with increased breast cancer risk, although it cannot be excluded that it is associated with a specific high–risk subgroup. Considering the low population frequency, much larger studies are needed to determine whether the latter is indeed the case.

The whole gene screen for *HOXB13* also identified c.649C > T (p.R217C) as a recurrent mutation in the Dutch population, which was predicted to be pathogenic. In both the discovery as well as the validation phase of the study, the mutation was more prevalent in familial breast cases than controls, but the association with breast cancer risk was not significant. Considering the wide CIs and the very low population frequency, there is a possibility that the study was underpowered and failed to detect the association. Evaluation of *HOXB13* p.R217C in a larger study or a population with a higher carrier allele frequency might therefore still be worthwhile to pursue. Since the p.G84E variant varies widely among different geographic populations (*i.e.* highest in North and West-Europeans and lowest in non-Europeans)^29^,^30^, this may also be the case for p.R217C. Interestingly, the p.R217C mutation had been described before among a few prostate cancer cases^29^,^31^, however, Xu *et al*. reported that p.R217C did not co-segregate with prostate cancer in the two families they identified^29^. Unfortunately, we were not able to perform informative segregation analysis in the present study as for only two families we had DNA available for two additional family members. In addition, we identified too little carriers of the mutation to say anything relevant regarding an excess of prostate cancer in their families as compared with non-carrier cases.

To conclude, none of the recurrent *HOXB13* mutations that we identified in the Dutch population were associated with breast cancer risk, although it may be worthwhile to evaluate p.R217C in a larger study or a population with a higher allele frequency.

## Methods

### Study population

The samples included in this study were from two Dutch breast cancer case-control studies: RBCS and ABCS-F. RBCS cases (N = 2,751) were selected from the database of the Clinical Genetics Centre at Erasmus University Medical Centre in Rotterdam, representing the Southwestern part of the Netherlands. Selected families included all families counselled between 1994 and 2014 that presented with at least two cases of female breast cancer or at least one case of female breast cancer and one case of ovarian cancer in first- or second-degree relatives. At least one of these two cases needed to be diagnosed before the age of 60. For each family, the youngest breast cancer patient who had been tested for *BRCA1* and *BRCA2* was assigned to be the index case and included in RBCS. Additionally, breast cancer cases were included that were diagnosed either before 40 years with unilateral breast cancer or before 50 years with bilateral breast cancer without having a first or second degree relative diagnosed with either breast or ovarian cancer. All cases and their tested relatives were negative for both *BRCA1* and *BRCA2* mutations. Median age of the RBCS cases was 44 years (range 18–92 years). The RBCS control population (N = 1,159) was geographically matched and included women from cystic fibrosis families who were either spouses of individuals at risk of being carrier of a *CFTR* mutation or individuals who were tested negative for a *CFTR* mutation and were counselled between 1996 and 2010. Median age of the RBCS controls was 41 years (range 10–97 years).

ABCS-F cases (N = 1,769) were selected from the linked databases of the Division of Diagnostic Oncology and the Tumor Registry of the Antoni van Leeuwenhoek hospital in Amsterdam^32^. We included female breast cancer patients of all ages (median age was 42 years (range 14–79 years)), without a pathogenic *BRCA1*/*2* mutation or unclassified variant, who were counselled in the Family Cancer Clinic and diagnosed and/or treated with cancer in the Antoni van Leeuwenhoek hospital in the period 1995–2012. For each family, only the youngest breast cancer patient who had been tested for *BRCA1* and *BRCA2* was included. ABCS-F controls (N = 1,968) are healthy women of all ages (median age was 49 years (range 18–69 years)) from the general population and were recruited through the blood bank.

All experiments were performed in accordance with the Code of Conduct of the Federation of Medical Scientific Societies in the Netherlands (http://www.fmwv.nl). The RBCS and ABCS-F studies were approved by the Medical Ethical Committes of the Erasmus Medical Center Rotterdam and the Netherlands Cancer Institute, respectively. All individuals gave written informed consent.

### PCR and Sanger sequencing

The entire *HOXB13* (RefSeq NM_006361.5) coding region was analyzed for sequence variations in 1,250 non-*BRCA1*/*2* familial breast cancer cases and 800 controls from RBCS (*i.e.* indexes from families counselled between 1995 and 2009 for cases and between 1996 and 2006 for controls) using PCR and Sanger sequencing. Twenty nanograms of DNA, extracted from peripheral blood, was PCR amplified in a final volume of 15 μl containing 1X GoTaq buffer (Promega, Madison, WI), 1.5 mM MgCl2, 200 μM dNTPs (GE Healthcare, Waukesha, WI), 1 μM of each primer and 0.75 U of GoTaq polymerase (Promega) using an ABI2720 thermal cycler (Thermo Scientific, Waltham, MA). First, the PCR reaction was incubated for 5 minutes at 94 °C, followed by 35 cycles of 94 °C for 30 seconds, 58 °C for 1 minute and 72 °C for 1 minute. The PCR reaction ended with a final extension at 72 °C for 5 minutes. Removal of dNTPs and primers before sequencing was done by ExoSAP-IT PCR Product Cleanup (Affymetrix, Santa Clara, CA). Briefly, 2.5 μl of PCR product was incubated with 0.5 μl of ExoSAP-IT and 1x GoTaq buffer in a final volume of 12.5 μl. Incubation took place in an ABI2720 thermal cycler for 15 minutes at 37 °C. Then enzymes were inactivated at 80 °C for 15 minutes before proceeding with Sanger sequencing. The sequencing reaction contained 2 μl of ExoSAP-it treated PCR product, 1 μl BigDye Terminator v3.1 reaction mix (Thermo Scientific), 1X BigDye Terminator sequencing buffer (Thermo Scientific) and 0.16 μM of sequencing primer in a final volume of 10 μl and was carried out in an ABI2720 thermal cycler according to the following protocol: 1 cycle of 96 °C for 2 minutes and 25 cycles of 96 °C for 30 seconds, 58 °C for 30 seconds and 72 °C for 2 minutes. Subsequently, the sequencing product was precipitated with absolute ethanol and 3M of NaAc, resuspended in 20 μl of Hi-Di formamide (Thermo Scientific), and ran on an ABI3130XL Genetic Analyzer (Thermo Scientific). Sequencing electropherograms were analyzed using Mutation Surveyor v3.20 software (Softgenetics, State College, PA). Sanger sequencing was successful for 96.2% of the samples and PCR and sequencing primer sequences for the two exons of the *HOXB13* gene are available in Supplementary Table 1.

### Taqman genotyping

Genotyping of the c.251G > A (p.G84E; rs138213197) and c.649C > T (p.R217C; rs139475791) mutations in the *HOXB13* gene was performed for all 7,647 DNA samples from RBCS and ABCS-F using custom-made Taqman genotyping assays (Thermo Scientific) on a Mx3000/3005P qPCR machine (Agilent Technologies, Santa Clara, CA). For genotyping p.G84E, 0.5X of Taqman genotyping assay and 0.5X of Taqman Genotyping Master Mix (Thermo Scientific) was added to 20 ng of genomic DNA in a final volume of 10 μl, whereas for p.R217C, 1X Taqman genotyping assay and 1X ABsolute qPCR Mix, low ROX (Thermo Scientific) was added to 20 ng of genomic DNA in a final volume of 10 μl. Cycling conditions were: 1 cycle of 10 minutes (for Taqman Genotyping Master Mix) or 15 minutes (for ABsolute qPCR Mix, low ROX) at 95 °C and 45 cycles of 15 seconds at 92 °C and 1 minute at 60 °C. The MxPro qPCR software v4.10 (Agilent) was used to visualize the genotyping results. The call rate of the genotyping assays was 97.9% for p.G84E and 98.1% for p.R217C, respectively, and Taqman assay design is specified in Supplementary Table 2. The accuracy of both genotyping assays was evaluated by comparing genotypes obtained from the 1,250 RBCS cases and 800 RBCS controls through Taqman genotyping with genotypes obtained from Sanger sequencing. For quality control, each 96-well plate included a wild-type and a heterozygous sample. Samples that were identified to be positive by either Sanger sequencing or in Taqman assays were independently confirmed by Sanger sequencing.

### Statistical analyses

The association of both *HOXB13* mutations (*i.e.* p.G84E and p.R217C) with breast cancer risk was evaluated by comparing the carrier allele frequency between cases and controls using either a χ^2^ test or a Fisher’s exact test (*i.e.* when the expected frequency ≤5 in any of the groups). Odds ratios and their 95% confidence intervals were calculated based on 2 × 2 table analysis of the cases and controls. All statistical tests were two-sided and *P*-values were considered statistically significant when smaller than 0.05.

## Additional Information

**How to cite this article**: Liu, J. *et al*. Recurrent *HOXB13* mutations in the Dutch population do not associate with increased breast cancer risk. *Sci. Rep.* **6**, 30026; doi: 10.1038/srep30026 (2016).

## Supplementary Material

Supplementary Information

## Figures and Tables

**Table 1:** Rare variants identified by PCR and Sanger sequencing of the *HOXB13* gene.

**Position**	**Nucleotide change**	**Amino acid change**	**Rs number**	**Carrier allele frequency**	
				**Controls**	**Cases**
5′UTR	c.1-6G > A			0/759 (0%)	1/1215 (0.08%)
Exon 1	c.251G > A	p.G84E	rs138213197	6/759 (0.79%)	4/1215 (0.33%)
Exon 1	c.328C > G	p.P110A		0/759 (0%)	1/1215 (0.08%)
Exon 1	c.330C > A	p.P110P	rs33993185	1/759 (0.13%)	0/1215 (0%)
Exon 1	c.332C > T	p.A111V		0/759 (0%)	1/1215 (0.08%)
Exon 1	c.569C > T	p.P190L		0/759 (0%)	1/1215 (0.08%)
Intron 1	c.601 + 49G > A		rs200606700	0/759 (0%)	1/1215 (0.08%)
Exon 2	c.649C > T	p.R217C	rs139475791	1/765 (0.13%)	6/1206 (0.50%)
Exon 2	c.803G > A	p.R268Q		0/765 (0%)	1/1206 (0.08%)
Exon 2	c.832G > T	p.V278L	rs200997384	1/765 (0.13%)	0/1206 (0%)
3′UTR	c.855 + 28C > A			3/765 (0.39%)	5/1206 (0.41%)

**Table 2:** Association of *HOXB13* p.G84E and p.R217C with breast cancer risk.

**Variant**	**Study**	**Carrier allele frequency**		**OR (95% CI)**	***P*****-value**
		**Controls**	**Cases**		
c.251G > A/p.G84E	RBCS	3/356 (0.84%)	9/1,465 (0.61%)		
	ABCS-F	13/1,944 (0.67%)	9/1,722 (0.52%)		
	Combined	16/2,300(0.70%)	18/3,187 (0.56%)	0.81 (0.41–1.59)	0.54
c.649C > T/p.R217C	RBCS	0/355 (0%)	5/1,473 (0.34%)		
	ABCS-F	2/1,933 (0.10%)	5/1,735 (0.29%)		
	Combined	2/2,288 (0.087%)	10/3,208 (0.31%)	3.57 (0.76–33.57)	0.14

OR: odds ratio, CI: confidence interval.
