# Supplementary material for: Recurrent HOXB13 mutations in the Dutch population do not associate with increased breast cancer risk
Source: Sci Rep. 2016 Jul 18;6:30026. doi: 10.1038/srep30026 (PMC4948019; doi:10.1038/srep30026)
Supplement: Supplementary Information [file srep30026-s1.pdf]

Recurrent *HOXB13* mutations in the Dutch population do not associate with increased breast cancer risk

Jingjing Liu, Wendy J.C. Prager – van der Smissen, Marjanka K. Schmidt, J. Margriet Collée, Sten Cornelissen, Roy Lamping, Anja Nieuwlaat, John A. Foekens, Maartje J. Hooning, Senno Verhoef, Ans M.W. van den Ouweland, Frans B.L. Hogervorst, John W.M. Martens, Antoinette Hollestelle.

Supplementary Data

Supplementary Table 1 PCR and sequencing primers for the *HOXB13* gene

	**Exon 1**	**Exon 2**
Forward primer	5'-GGCCCCTGCGTCTCTTG-3'	5'-CTGGTATGCTTTGGAGGAAC-3'
Reverse primer	5'-GAGGAGCACCAAGCTCATC-3’	5'-GGCCGCTCCTGAGGAAC-3'

Supplementary Table 2 Primers and reporter probes for *HOXB13* Taqman genotyping assays

	***HOXB13* c.251G>A (p.G84E)**	***HOXB13* c.649C>T (p.R217C)**
Forward primer	5'- GGAGCCGCCAAAGCAAT -3'	5'- CCTTTTCCTCCCTCCCTTTCAC -3'
Reverse primer	5'- CGAGCTCCGGGACACT-3’	5'- TCCAGCTCCCGCAACTG-3’
VIC reporter probe	5'- CCGCCTCCAAAGTA -3'	5'- CGTTTCTTGCGGCCGC -3'
FAM reporter probe	5'- CCGCCTTCAAAGTA -3'	5'- CGTTTCTTGCAGCCGC -3'
